# The Etiology of Disease of the Bile-Bearing Passages

**Published:** 1917-03

**Authors:** C. O. Harper

**Affiliations:** Fort Worth, Texas


### TEXAS MEDICAL JOURNAL

DR. F. E. DANIEL,
Founder.

MRS. F. E. DANIEL,
Publisher and Managing
Editor.

PUBLISHED MONTHLY.
Subscription:
$1.00 a Year.

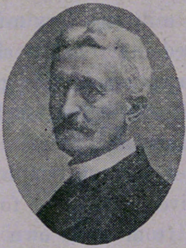

Established July,
1885.

VOL. XXXII No. 9
AUSTIN,
MARCH, 1917.

The publisher is not responsible for views of contributors.

*"All of good the past hath had,
Remains to make our own time glad."*—WHITTIER.

### Original Articles.

### The Etiology of Disease of the Bile-Bearing Passages.

BY C. O. HARPER, M. D., FORT WORTH, TEXAS.

For the sake of system, probably may be divided into anatomic, physiologic, chemical and bacteriologic influences, each acting with another or severally. This, however, is nothing new except probably we have not given as much attention and credit to the physiologic chemistry of the liver secretions and its cellula activity as detoxicating agents. For in this gland chemicals are transformed, bacteria and their toxins attenuated; in fact, it is a filter plant not to be disregarded. In order to understand the multiplicity of causes best, we find it necessary to recall why there is, as a rule, no infection, and that such infection is the exception. From experiments we have learned that bile is not as much of an antiseptic as we once thought, but that the liver cells by their specific action, as above stated, continuously attenuate the constant systemic infection, especially from the alimentary tract by several routes. These cells are so highly organized and efficient that they not only constantly detoxicate the blood and lymph (i. e., acting watchman of the portal system) but actually can reproduce liver in case of loss of part of this gland.. Second to this cytologic function probably stands the importance of good drainage. Gravity has its influence in the main, except when the depth of the pelvis of the gall bladder becomes exaggerated, or when adhesions obstruct the ducts. The amount of bile seems to play an important rôle which, if great, causes the same to be thinner, and, of course, to flow faster and more constantly, giving a more perfect drainage. I can readily see that for the above stated condition to obtain there must be a normal blood supply to the liver and its ducts, a lack of which militates very much against their normal functions, consequently sedentary habits and imperfect circulation are indirect causes of infection of the gall bladder and hepatic ducts, explaining why those who lead such a life get cholesystitis and the sequence cholelithiasias.

Hepatic statis of either bile or lymph are no doubt frequent causes of trouble. The liver cells suffer for well oxygenated blood, and becomes intoxicated from their own effete matter by reason of such abnormal physiology. Proper cardiac action and hepatic activities are necessary for rightful bile functions. Someone has stated that bile secretion is governed more by chemical reactions than by innervation and circulation. If this be true, the quantity and character of food should be very influential. I believe it is a conceded fact that the normal stimulus of bile secretions are the bile salts, which explain the automatic function of this organ. Whether the percentage of bile salts has anything to do with the precipitation of cholesterine, I believe is yet uncertain. It would seem from a chemical standpoint, as the demand of the system arises, if not too sudden or excessive, are neutralized by the liver function.

Having described or recalled those conditions that prevent infection, we are, I believe, prepared to understand the causes of same.

Taking up, first, the anatomical reasons, the bile radicals are very numerous and small, making it necessary for discharge of a given amount in about a given time; the circulation being a dual one, the venous and arterial and in the liver—being slow and under low pressure, these conditions, if much disturbed from any cause, must defeat the normal and relative functions of all the bile-bearing area.

Pathologic physiology as a cause, an imperfect circulation of blood and lymph especially through this area, often due to cardiac lesions (chronic), alcoholism, excessive eating, visceral lues, by inducing circulatory stasis of stomach, duodenum and their attendant, catarrhal condition, eventually leads to an involvement of the bile ducts and gall bladder. The consequent excessive secretions of mucus which serve, no doubt, a splendid culture media for pathogenic bacteria, thus promoting their pathogenicity. Knowing, as we do, that the bile normally contains such organisms, that excessive eating of carbohydrates as a special etiologic influence has not yet been proven, which was supposed to increase the production of bile salts. I believe overeating and the lack of oxidation of the same due to extreme sedentary habits, by inducing chronic hyperaemia of the alimentary tract and liver is the chief indirect cause of pathology of the biliary tract.

Of the abnormal chemical reactions that precipitate or make possible such abnormal anatomy, I am prepared to say very little, not having done much physiologic experimentation; however, recounting the fact that the liver with the animentary tract is the preparatory and transforming laboratory of the entire organism, lends to this phase of the subject a possible etiology not yet explained.

The direct or immediate cause is well known by every one and due to bacteria. The colon bacilli, bacillus of Eberth and other pyogenic cocci.

It is interesting to note that after infection has occurred that in more than fifty per cent of cases where cultures were attempted they proved to be sterile. Just at what time this obtains has not yet been determined.

The clinical histories of infections of this region are often traceable to ulcerating hemorrhoids and their infected thrombi, duodenal and stomach ulcers, peritoneal lymphatic involvement, the last named, however, being an infrequent portal.

The lack of lymph glands and few lymph vessels probably are two of the many reasons of limited protection to this area, especially when normal bile drainage is interfered with.

It is apparent, I think, that the liver often strikes the balance between disease and health and that the stealthiness of the pathology of this tract is indeed a bugbear to the diagnostician and surgeon alike. As to the cause of malignancy, it appears that the history teaches that irritants are especially productive of this condition, and in this anatomic region gallstones and infection are the chief offenders, probably exciting to excessive activity the latent cells. This occurring in those who are susceptible to cancer, except in those cases where the malignancy primarily is of the bowels or stomach and metastatic in the biliary tract.

---

Members of the Tarrant County Medical Society at their monthly meeting held in Fort Worth on February 16th were told of the American Red Cross call for volunteers to serve in the event of war. Copies of the call were sent out from national headquarters to every Red Cross chapter in the country.

